# Efficacy and safety of oral Guanxinshutong capsules in patients with stable angina pectoris in China: a prospective, multicenter, double-blind, placebo-controlled, randomized clinical trial

**DOI:** 10.1186/s12906-019-2778-z

**Published:** 2019-12-11

**Authors:** Yang Li, Lei Zhang, Shuzheng Lv, Xiaozeng Wang, Jian Zhang, Xiaoxiang Tian, Yan Zhang, Bojun Chen, Dayue Liu, Jie Yang, Peikang Dong, Yunzhong Xu, Yingmin Song, Junling Shi, Lian Li, Xuechang Wang, Yaling Han

**Affiliations:** 1 Department of Cardiology, General Hospital of Northern Theater Command, 83 Wenhua Rd, Shenyang, 110016 Liaoning Province China; 2 Department of Cardiology, Beijing Anzhen Hospital, Capital Medical University, 2 Anzhen Rd, Beijing, 100029 China; 3 Affiliated Hospital of Liaoning Traditional Chinese Medicine University, 33 Beiling St, Shenyang, 110032 Liaoning Province China; 4 Guangdong Traditional Chinese Medicine Hospital, 111 Dade Rd, Guangzhou, 510120 Guangdong Province China; 5 Xuzhou Center Hospital, 199 Jiefang Rd, Xuzhou, 221009 Jiangsu Province China; 6 Huanggang Center Hospital, 16 Kaopeng St, Huanggang, 438000 Hubei Province China; 7 Affiliated Hospital of Weifang Medical University, 2428 Yuhe Rd, Weifang, 261031 Shandong Province China; 8 Second Affiliated Hospital of Shandong Traditional Chinese Medicine University, 1 Jingba Rd, Jinan, 250001 Shandong Province China; 9 Luohe Hospital of Chinese Medicine, 649 Jiaotong Rd, Luohe, 462000 Henan Province China; 10 Tangshan Hospital of Chinese Medicine, 6 Kangzhuang Rd, Tangshan, Hebei Province China; 11 Shijiazhuang First Hospital, 36 Fanxi Rd, Shijiazhuang, 050011 Heibei Province China; 12 Yunnan Third Hospital, 292 Beijing Rd, Kunming, 650011 Yunnan Province China

**Keywords:** Angina, Randomized clinical trial, Exercise tolerance test, Quality of life, Guanxinshutong

## Abstract

**Background:**

To assess the efficacy and safety of oral Guanxinshutong (GXST) capsules in Chinese patients with stable angina pectoris (SAP) in a prospective, multicenter, double-Blind, placebo-controlled, randomized clinical trial (clinicaltrials.gov Identifier: NCT02280850).

**Methods:**

Eligible patients were randomized 1:1 to the GXST or placebo group. Current standard antianginal treatment except for nitrate drugs was continued in both groups, who received an additional 4-week treatment of GXST capsule or placebo. Primary endpoint was the change from baseline in angina attack frequency after the 4-week treatment. Secondary endpoints included the reduction of nitroglycerin dose, score of Seatntle Agina Questionnaire, exercise tolerance test defined as time to onset of chest pain and ST-segment depression at least 1 mm greater than the resting one.

**Results:**

A total of 300 SAP patients from 12 centers in China were enrolled between January 2013 and October 2015, and they were randomly divided into the GXST group and the placebo group (150 patients in each group). Of whom, 287 patients completed the study (143 patients in the GXST group, 144 patients in the placebo group). The baseline characteristics of the two groups were comparable. After 4-week treatment with GXST capsules, the number of angina attacks and the consumption of short-acting nitrates were significantly reduced. In addition, the quality of life of patients were also substantially improved in the GXST group. No significant differences in the time of onset of angina and 1-mm ST segment depression were noted between the two groups. 7 patients (4.1%) in the GXST group and 3 patients (2.1%) in the placebo group reported at least one adverse event, respectively.

**Conclusions:**

GXST capsules are beneficial for the treatment of SAP patients.

## Background

Stable angina pectoris (SAP) is one of the most common subtypes of coronary heart disease, and it affects approximately 54 million patients worldwide [1, 2]. According to guidelines published by the health authorities of the United States [3], Canada [4], Europe [5] and China [6], the current treatment for SAP relies on the use of anti-ischemic drugs, such as nitrates, β-blockers, calcium channel blockers (CCB), angiotensin converting enzyme inhibitors (ACEI)/angiotensin II receptor blockers (ARB), and is often combined with secondary prevention measures such as lipid lowering and anti-platelet drugs to prevent myocardial infarction and death, reduce the symptoms of ischemic attack and improve the quality of life (QOL) of patients [7, 8]. In addition, patients with symptomatic angina are often treated with both traditional Chinese medicine and western medicine in China.

Traditional Chinese medicine has been used in the treatment of chronic heart disease for more than 2000 years in China [9–11]. The components of the traditional herb and Mongolian medicine, Guanxinshutong (GXST) capsules, are *Choerospondias axillaris*, *Salvia miltiorrhiza*, *Syzygium aromaticum*, *borneol* and *concretio silicea bambusae*. Animal experiments in rats have shown that GXST capsules can increase coronary blood flow, improve myocardial oxygen supply, and regulate vascular compliance [12, 13]. Moreover, our previous studies have demonstrated that GXST capsules can limit myocardial infarct size through inhibition of inflammatory response and apoptosis in rats [12, 13]. Furthermore, a clinical observational study has shown that GXST capsules are effective and safe for the treatment of SAP patients [14]. However, the efficacy of the drug has not been established by well-designed randomized clinical trials in China.

To our knowledge, this is the first prospective, multicenter, double-blind, placebo-controlled, randomized clinical trial assessing the efficacy and safety of orally administered GXST capsules in SAP patients by comparing the effects of GXST capsules on antianginal effect, exercise tolerance and safety in SAP patients in China .

## Methods

### Study design

**Fig. 1:**
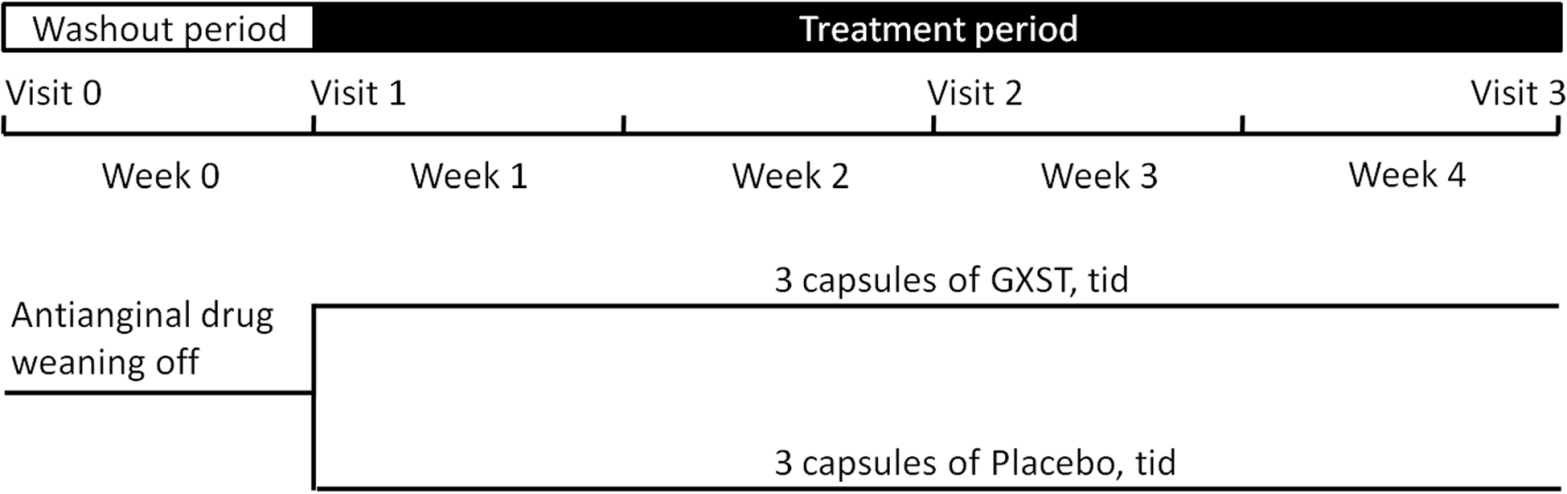
Study program for each subject. GXST = guanxinshutong capsule

This was a phase IV double-blind, randomized, and placebo-controlled study conducted at 12 centers in China between January 2013 and October 2015. Neither the investigator nor the subject was aware of what treatment the subject received. The disposition of patients is summarized in Fig. 1. The study was designed and conducted in accordance with the requirements of Good Clinical Practice (GCP) and the Declaration of Helsinki. The study has been reviewed and approved by the Ethics Committee of General Hospital of Northern Therter Command (K (2013) 11). Then the study protocol was reviewed and approved by the Institutional Review Board of each medical center involved. All patients provided written informed consent prior to the study.

### Enrollment criteria

Patients aged 18 to 75 years old were eligible to participate in the study if they had diagnosis of coronary artery disease (CAD) with SAP, as documented by at least one of the followings: 1) previous diagnosis of myocardial infarction (more than 6 months ago); 2) coronary angiography or computerized tomography coronary angiography showed at least one major coronary branch stenosis more than 50%; 3) underwent coronary revascularization (percutaneous coronary intervention (PCI) or coronary-artery bypass grafting) more than 3 months ago; 4) typical angina symptoms or positive exercise tolerance text (ETT). Major exclusion criteria included acute coronary syndrome, left main CAD regardless of whether revascularization was performed or not, aortic stenosis (moderate to severe), hypertrophic obstructive cardiomyopathy, congestive heart failure, echocardiographic left ventricle ejection fraction less than 40%, uncontrolled severe hypertension, complete left bundle branch block, Wolff-Parkinson-White syndrome, presence of ventricular paced rhythm, as well as liver and renal dysfunction.

### Randomization and treatment

Patients were randomly assigned without stratification to receive GXST capsules or placebo in a 1:1 ratio using sealed envelopes. The physicians, patients, evaluators, and statistician were unaware of the random allocation. Treatment in the double-blind phase was continued until disease progression, adverse events (AEs), withdrawal of consent, or primary analysis. There were two times of unblinding during the study. For the first time of unblinding, the plan for the statistical analysis was completed before the database was locked and unblinded, then the second time of unblinding was carried out, and all patients were unblinded. All the patients included in this study received GXST capsule treatment or placebo treatment. In addition to nitrate drug treatment, the standard antianginal treatment was also allowed as a basic treatment, including aspirin, β-blockers, CCB, lipid-lowering statin drugs and ACEI/ARB. All of these treatments will be recorded in patients’ medical records as well as their case report forms (CRFs) in detail.

Each patient was asked to make a total of 3 study visits on Day − 7, 0 and 28 (±2) after randomization and treatment initiation. At the screening visit (Day − 7), demographic data, medical history, and concomitant medications were recorded, and physical examination was performed. Patients meeting the eligibility criteria entered the double-blind portion of the study. At baseline (Day 0), the number of average angina attacks per week were recorded, and the patients in each group underwent an ETT (modified Bruce Protocol) before being randomized to receive either 3 capsules of 0.3 g GXST or placebo three times daily for 4 weeks. The placebo was composed of *Corn Starch*, *Silica*, *Caramel color*, *Sunset Yellow*, Mixed Powder for GXST capsule and ethanol, which were then filled into the capsules. The appearance and smell of placebo are the same as that of GXST capsules. At the end of 4-week double-blind treatment, patients were instructed to return for clinic visit and all assessments including the number of average angina attacks per week, physical examination, resting electrocardiogram and ETT were repeated.

A nitroglycerin tablet (0.5 mg per tablet, manufactured by Beijing Yimin Pharmaceutical Group Co., Ltd.) is allowed if the patient has an attack of angina, and the patient will be asked to record the doses and times of administration in detail, and these informations will be collected by the investigator at the next follow-up visit. If the symptoms of angina are not relieved after 3 doses of nitroglycerin, the patient will be asked to go to the hospital for further treatment. If the patient has other underlying diseases, the concomitant drugs considered necessary for the treatment of these diseases are allowed to be used during the study period, and these drugs should be recorded in the patients’ medical records and CRFs in detail. However, no other Chinese herbal medicines or Chinese patent medicines with similar effects like GXST capsules are allowed to be used during the study period.

### Endpoints and definitions

The primary outcome in this study is the change from baseline in angina attack frequency at 4 weeks of treatment. The primary endpoint will be measured 1 day before the inclusion and the 1st day after 4 weeks post-inclusion. The secondary outcomes include the following three items: 1) reduction of nitroglycerin dose; 2) score of Seatntle Agina Questionnaire (SAQ) [15, 16]; 3) positive ETT was defined as the time of onset of chest pain, and ST-segment depression at least 1 mm greater than the resting one, with or without limiting angina. For the overall efficacy assessment, the number of weekly anginal attacks were categorized according to the guideline for clinical trial of cardiovascular drugs in China [17]. A > 80% reduction in the number of angina attacks and nitroglycerin consumption after treatment was defined as highly effective, a 50–80% reduction was defined as effective, and a < 50% reduction was defined as no effically. QOL was assessed at baseline and 4 weeks after the procedure with the SAQ. The SAQ is a disease-specific instrument that quantifies five dimensions of QOL that are important to patients with angina and CAD, including physical limitations due to CAD, angina stability, angina frequency, satisfaction with treatment, and disease perception. All SAQ domains are scaled from 0 to 100 points, with higher scores indicating better QOL. All patients given double-blind drug with documented follow-up were included in the safety evaluation. Patients were observed and questioned at each visit about the occurrence of adverse events.

### Statistical analysis

SPSS version 19.0 was used for statistical analysis. Results were presented as mean ± standard deviation (SD). All analyses were done by intention to treat. Missing data were not replaced. Outcomes data for the primary and secondary endpoints were compared as binary proportions. Categorical variables were compared using the Chi-squared test or Fisher’s exact test, and continuous data were compared using the t test or one-way analysis of variance. *P* < 0.05 was considered as the level of significance.

## Results

### Patient characteristics and baseline comparison

**Fig. 2:**
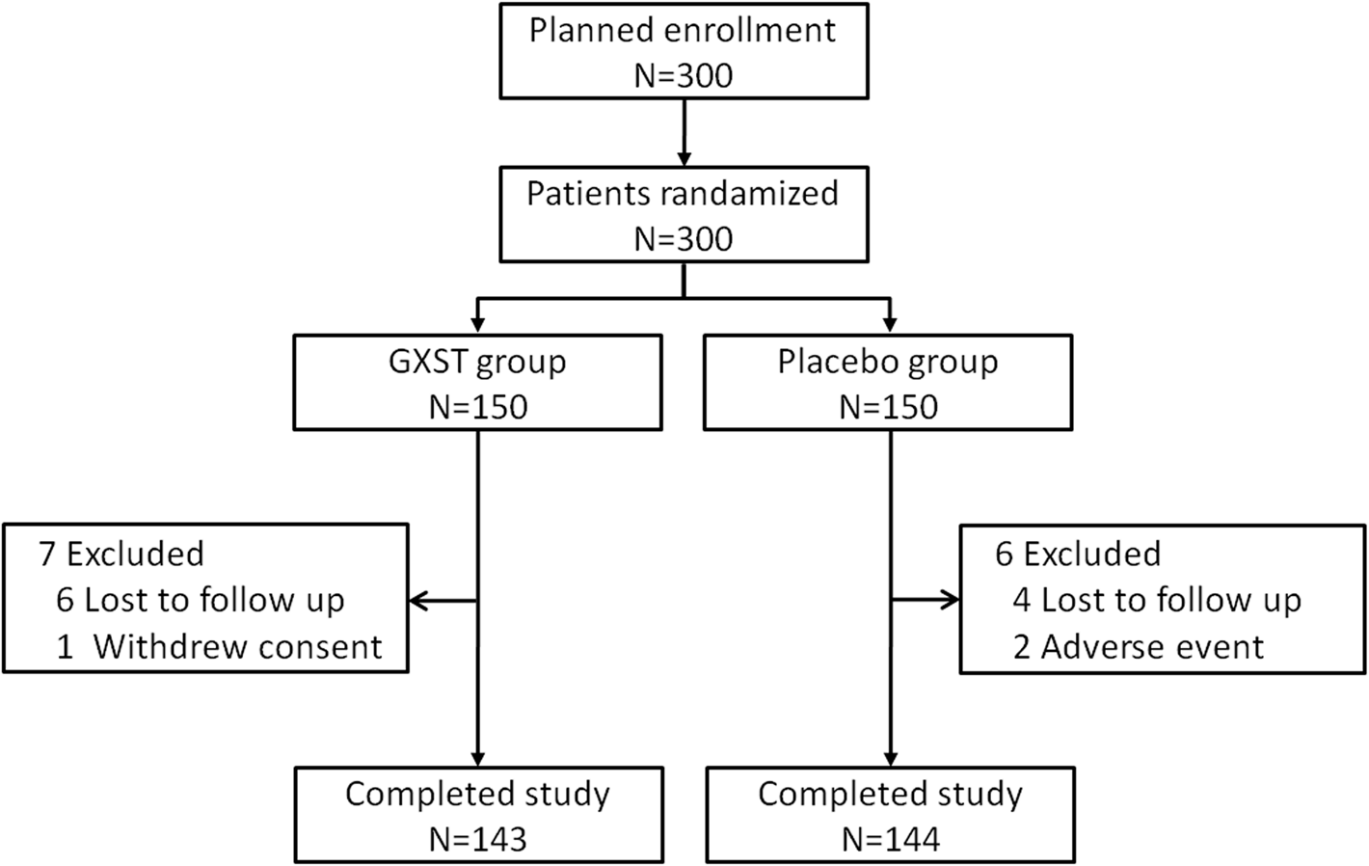
Diagram of patient flow in the trial. GXST = guanxinshutong capsule

Signed informed consent was given by 300 SAP patients from 12 centers in China between January 2013 and October 2015. Of the 300 randomized patients there were 150 patients in the GXST group and 150 patients in the placebo group; 287 patients completed the study (143 patients in the GXST group, 144 patients in the placebo group) (Fig. 2). Before the primary endpoint analysis, 1 patient in the GXST group discontinued the study due to withdrawal of consent; 6 patients in the GXST group and 4 patients in the placebo group were lost to follow-up; 2 patients in the placebo group dropped out because of the onset of palpitation.

Baseline characteristics were comparable in age, gender, median body mass index, and smoking status between the two groups. At the time of enrolment, all the patients in the two groups were taking one or more types of standard anti-angina therapies such as ACEI/ARB, β-blocker, CCB, aspirin/anti-platelet drugs and lipid-lowering drugs. Detailed data of patient characteristics, medications and medical history were summarized in Table 1.

**Table 1:** Baseline Characteristics and Concomitant Medications

Characteristic	Treatment group		*P* value
	GXST (*n* = 143)	Placebo (*n* = 144)	
Demographics			
Gender (M/F)	(73/70)	(82/62)	0.16
Age (years)	56.6 ± 8.1	55.3 ± 8.3	0.15
Height (cm)	166.6 ± 7.7	167.5 ± 7.7	0.31
Weight (kg)	68.9 ± 10.2	68.9 ± 9.8	1.00
HR (beats/min)	71.2 ± 9.3	70.7 ± 9.3	0.64
SBP (mmHg)	128.8 ± 9.7	129.0 ± 9.2	0.90
DBP (mmHg)	80.9 ± 7.2	80.1 ± 7.7	0.41
Baseline characteristics			
Angina frequency (no. of events per week)	9.4 ± 3.2	9.0 ± 3.0	0.32
NTG consumption (no. of pills per week)	5.8 ± 3.5	5.5 ± 3.0	0.49
SAQ score Physical limitations Angina stability Angina frequency Treatment satisfaction QOL	46.8 ± 10.6 51.8 ± 11.1 51.8 ± 8.7 36.9 ± 20.0 46.5 ± 18.2 44.4 ± 19.5	48.2 ± 12.0 54.1 ± 12.0 51.0 ± 9.1 43.6 ± 21.1 47.5 ± 19.2 45.0 ± 20.4	0.28 0.08 0.42 0.09 0.64 0.81
ETT Time to onset of chest pain (s)	430.24 ± 177.2	456.42 ± 188.72	0.71
Time to 1 mm ST-depression (s)	235.95 ± 153.11	237.69 ± 174.34	0.96
Concomitant medication			
β-blocker	10 (7.0%)	15 (10.4)	0.15
ACEI	3 (2.1%)	0 (0.0%)	0.06
ARB	10 (7.0%)	8 (5.6%)	0.31
CCB	10 (7.0%)	9 (6.3%)	0.40
Stains	126 (88.1%)	125 (86.8%)	0.38
Anti-platelet agents	138 (96.5%)	140 (97.2%)	0.41

Each value represents the mean ± SD or the number (%)

GXST, Guanxinshutong capsule; ACEI, angiotensin I converting enzyme inhibitors; ARB, angiotensin II receptor blockers; CCB, calcium channel blockers, ETT, exercise treadmill test; QOL, quality of life; SAQ, Seatntle Agina Questionnaire; SBP, systolic blood pressure; DBP, diastolic blood pressure; HR, heart rate

### Primary endpoint

The number of anginal attacks at week 0 was defined as the baseline value. GXST capsules significantly decreased the number of attacks in comparison with placebo at week 4 (Table 2). The average number was 9.4 per week at the beginning of the study, but was significantly reduced to 2.5 attacks per week after the GXST therapy (*P* = 0.02). However, there was no difference between the baseline and treatment in the placebo group (*P* = 0.44). In the GXST group, GXST capsules were highly effective in 25.2% patients, effective in 51.0% patients, and there was no efficacy in 23.8% patients; in the placebo group, GXST capsules were only highly effective in 12.5% patients, effective in 33.3% patients, and there was no efficacy in most of the patients (54.2%).

**Table 2:** Primary efficacy evaluation

	Treatment group		P value
	GXST (*n* = 143)	Placebo (*n* = 144)	
Angina attack frequency (times/week)			
Before treatment	9.4 ± 3.2	9.0 ± 3.0	0.44
After 4-week treatment	2.5 ± 1.7	7.5 ± 2.2	0.02
Highly effective	36 (25.2%)	18 (12.5%)	0.005
Effective	73 (51.0%)	48 (33.3%)	0.002
No efficacy	34 (23.8%)	78 (54.2%)	< 0.001

Each value represents the number (%)

GXST, Guanxinshutong capsule

### Secondary endpoints

The nitroglycerin consumption at week 0 was defined as the baseline value. GXST capsules significantly decreased the nitroglycerin consumption in comparison with placebo at week 4 (*p* < 0.0001). In the GXST group, the nitroglycerin consumption was reduced by more than 80% in 39.9% patients after treatment, a 50–80% reduction was found in 41.3% patients, and a < 50% reduction was only found in 18.8% patients; in the placebo group, a > 80% reduction of nitroglycerin consumption was only found in 8.3% patients after treatment, a 50–80% reduction was in 41.7% patients, and a < 50% reduction was in most of the patients (50%).

At week 0, there were no significant differences in the SAQ score and subscales between the two groups. As shown in Table 3, highly significant improvements were found in the SAQ score, angina stability, angina frequency, treatment satisfaction, and disease perception (*p* < 0.0001 for all) in the GXST group, while there were no significant differences in physical limitations between the two groups (60.1 ± 10.5 vs. 58.0 ± 11.7, *p* = 0.11).

**Table 3:** Secondary efficacy evaluation

	Treatment group		P value
	GXST (n = 143)	Placebo (n = 144)	
Nitroglycerin consumption			
Highly effective	57 (39.9%)	12 (8.3%)	< 0.0001
Effective	59 (41.3%)	60 (41.7%)	0.52
No efficacy	27 (18.8%)	72 (50.0%)	< 0.0001
SAQ score	65.6 ± 10.6	56.3 ± 13.4	< 0.001
Physical limitations	60.1 ± 10.5	58.0 ± 11.7	0.11
Angina stability	79.6 ± 19.4	66.0 ± 22.6	< 0.001
Angina frequency	59.7 ± 20.1	49.5 ± 20.0	< 0.001
Treatment satisfaction	65.6 ± 14.3	54.5 ± 19.0	< 0.001
QOL	62.9 ± 16.1	53.4 ± 19.1	< 0.001
ETT			
Time to onset of chest pain (s)	517.6 ± 165.2	493.2 ± 152.9	0.81
Time to 1 mm ST-depression (s)	219.7 ± 172.2	257.7 ± 183.6	0.37

Each value represents the mean ± SD or the number (%)

ETT, exercise treadmill test; GXST, Guanxinshutong capsule; QOL, quality of life; SAQ, Seatntle Agina Questionnaire

For the pretreatment ETT, patients in both the GXST and placebo groups had no difference in the time of onset of chest pain (430.24 ± 177.2 s vs. 456.42 ± 188.72 s, *p* = 0.71, Table 3) and 1 mm ST-depression (235.95 ± 153.11 s vs. 237.69 ± 174.34 s, *p* = 0.96, Table 3). After the 4-week treatment, there was also no significant difference in the time of onset of chest pain (GXST group vs. placebo group, 517.6 ± 165.2 s vs. 493.2 ± 152.9 s, *p* = 0.81, Table 3) and 1 mm ST-depression (GXST group vs. placebo group, 219.7 ± 172.2 s vs. 237.7 ± 174.3 s, *p* = 0.37, Table 3) between the two groups.

### Safety

Safety population included all patients (*n* = 287) who received study drug (basic treatment plus GXST capsule or placebo). As seen in Table 4, similar rates of AEs were observed in the GXST (4.9%) and placebo (2.1%) groups (*p* = 0.165) during the study. Five AEs reported in the GXST group were considered to be related to the study drug with the exception of urinary tract infection in one patient and upper respiratory tract infection in one patient. In the GXST group, gastrointestinal discomfort was the most frequent adverse event (3.5%, 5/143).

**Table 4:** Vital signs and most frequent treatment adverse events

	GXST (n = 143)	Placebo (n = 144)	Total (n = 287)
Vital signs			
HR (beats/min)	71.2 ± 9.3	69.6 ± 8.9	70.2 ± 9.0
SBP (mmHg)	127.5 ± 9.3	127.5 ± 7.9	127.5 ± 8.5
DBP (mmHg)	80.9 ± 6.3	80.7 ± 6.5	80.9 ± 6.3
Adverse events			
Fever	0 (0.0%)	1 (0.7%)	1 (0.3%)
Hypertension	0 (0.0%)	1 (0.7%)	1 (0.3%)
Palpitation	0 (0.0%)	1 (0.7%)	1 (0.3%)
Urinary tract infection	1 (0.7%)	0 (0.0%)	1 (0.3%)
Upper respiratory tract infection	1 (0.7%)	0 (0.0%)	1 (0.3%)
Gastrointestinal discomfort	5 (3.5%)	0 (0.0%)	5 (1.7%)
Total	7 (4.9%)	3 (2.1%)	10 (3.5%)

Each value represents the mean ± SD or the number (%)

GXST, Guanxinshutong capsule

## Discussion

In this propective, multicenter, double-Blind, placebo-controlled, randomized clinical trial conducted in China, the results clearly showed that 4 weeks of adjunctive therapy with GXST capsules significantly reduced the number of anginal attacks in SAP patients. Moreover, the effects of GXST capsules were also assessed and demonstrated by secondary endpoints associated with the reduction of nitroglycerin consumption, as well as the improvement of SAQ score and subscales, although no significant differences in the time of onset of chest pain and 1 mm ST-depression in ETT were identified between the GXST and the placebo groups. Our results demonstrated that GXST capsules improve the clinical outcome of current standard therapies for SAP even with commonly used secondary prevention medicines.

The incidence of ischemic heart disease in China has increased despite aggressive management. SAP is a chronic disease condition that is treated to abolish or minimize symptoms, improve QOL, and reduce long-term morbidity and mortality [18–20]. Chinese herbal medicines have been widely used as an adjunct to western medicines, including nitrates, ACEI/ARB, β-blockers, CCB, aspirin/anti-platelet drugs and lipid-lowering drugs in treating angina in China [9–11]. However, there is still a knowledge gap to clearly establish evidence that Chinese herbal medicines are effective in improving the outcomes of angina patients.

The components of GXST capsule are *Choerospondias axillaris*, *Salvia miltiorrhiza*, *Syzygium aromaticum*, *borneol* and *concretio silicea bambusae*. In rat myocardial ischemia/reperfusion model, GXST capsules can reduce myocardial ischemia and infarct size, inhibit the increase of serum creatine phosphokinase activity, prevent cardiomyocyte apoptosis, decrease serum inflammatory cytokine levels following myocardial ischemia/reperfusion injury, increase coronary blood flow and myocardial oxygen supply [12, 13]. GXST capsules also inhibited the formation and progression of atherosclerotic plaque and stabilized the unstable plaque through down-regulating the MMP-9 expression in ApoE^−/−^ mice model [21]. Moreover, GXST capsule had a potential inhibitory effect on platelet aggregation stimulated by ADP and Thrombin in rats, thereby having a definite therapeutic effect against thromboembolic disease [22]. Screening and analysis of key active constituents in GXST capsule using mass spectrum and integrative network pharmacology, 12 active compounds and 33 targets were found to have a role in the treatment of cardiovascular diseases, and 4 main active ingredients, including *protocatechuic acid*, *cryptotanshinone*, *eugenol* and *borneol* were selected to verify the effect on calcium signaling system in cardiomyocyte injury induced by hypoxia and reoxygenation, providing an understanding of the therapeutic effect of GXST capsules [23]. To further strengthen those data, we now decided to design a clinical trial as this might give a more realistic picture of the everyday use of the drug.

In the present study, we found that the number of weekly anginal attacks and nitroglycerin consumption with GXST capsule treatment were significantly reduced in comparison with placebo. The magnitude of antianginal benefit observed in the trial is similar to that observed in other antianginal trials using conventional agents [19], in which patients with minimal or moderate anginal symptoms receiving a maximum recommended therapeutic dose of a β-blocker, an additional reduction was observed when the β-blocker was combined with a CCB titrated to its maximal tolerated dose [24]. Of note, however, the benefits achieved by combining the β blocker and a CCB were associated with significant undesirable changes in hemodynamics. The use of GXST capsules may allow for more optimal anti-ischemic effect without excessive adverse effects on heart rate and blood pressure.

With regard to exercise capacity, there were no statistically significant differences in the time of onset of angina and 1-mm ST segment depression between the GXST and placebo groups. However, an increase in the duration of the two key efficacy parameters at peak and at trough was found in both GXST and placebo groups, indicating that both GXST and placebo generally had a favorable effect on ETT durations. These results may indicate a training effect, but are more likely a strong placebo effect which has been documented previously in SAP patients [25].

The SAQ, designed to be applicable across sex, race, and socio-economic status, is a 19-item self-administered questionnaire measuring health status in patients with ischemic heart disease across 5 domains: physical limitation, angina stability, angina frequency, treatment satisfaction, and QOL [15, 16]. One previous review on antianginal drugs, such as long-acting nitrates, β-blockers, and CCBs, and their impact on QOL failed to show a significant effect over placebo [26]. This study furthers the understanding of the benefits of GXST on QOL. We had documented the improvement in all SAQ domains. Interestingly, at 4-week follow-up, GXST treatment was associated with improvements in angina frequency, angina stability, treatment satisfaction, and QOL domains except for physical limitations when it was compared with placebo. Taken together, all these results suggested that GXST capsules are effective in symptom alleviation and disease modification in SAP patients.

In general, a few patients in the GXST group experienced treatment AEs. However the AEs observed in the GXST group were in line with the established safety profile of GXST capsules. Previous studies have reported that gastrointestinal discomfort is the most common adverse event. The safety data of GXST capsules in this study is comparable to previous reports and no new safety concern is generated.

Limitations of this study included a relatively short treatment duration-4 weeks. Longer term efficacies of GXST treatment still need to be assessed. In addition, there was only about 35% of the patients in the GXST and placebo groups taking one or more types of standard anti-angina therapies such as ACEI/ARB, β-blocker, aspirin/anti-platelet drug and lipid-lowering drugs. Whether GXST coadministered with other classic antianginal agents such as β-blockers and CCBs will be useful in very symptomatic SAP patients remains need to be confirmed. Well designed placebo-controlled trials are needed to address this issue.

## Conclusions

GXST capsules were effective and well tolerated in SAP patients investigated in the trial. This resulted in a substantial reduction in the number of angina attacks and consumption of short-acting nitrates. Patients QOL was also substantially improved. Therefore, GXST capsules are beneficial for the treatment of SAP patients.

## Data Availability

All data generated or analysed during this study are included in this published article.

## Abbreviations

ACEI: angiotensin converting enzyme inhibitors
AEs: adverse events
ARB: angiotensin II receptor blockers
CCB: calcium channel blockers
CRFs: case report forms
ETT: exercise tolerance test
GXST capsule: Guanxinshutong capsule
QOL: quality of life
RCT: randomized clinical trial
SAP: stable angina pectoris
SAQ: Seatntle Agina Questionnaire
